# Analysis of lymph node spread and its prognostic significance in ampullary adenocarcinoma: A retrospective study

**DOI:** 10.3389/fsurg.2022.901615

**Published:** 2022-08-26

**Authors:** Zheng-Yun Zhang, Jiao Guan, Xin-Ping Wang, Di-Si Hao, Zun-Qiang Zhou

**Affiliations:** ^1^ Department of Surgery, Shanghai Jiao Tong University Affiliated Sixth People's Hospital, Shanghai, China; ^2^ Department of Surgery, Heilongjiang Provincial Hospital Affiliated to Harbin Institute of Technology, Harbin, China

**Keywords:** ampullary adenocarcinoma, metastasis, lymph node, prognosis, survival

## Abstract

**Background:**

Nodal status is a vital prognostic factor for ampullary adenocarcinoma. This study was designed to evaluate the clinical significance of the positive nodes in this disease.

**Methods:**

Data from 110 patients who underwent curative pancreatoduodenectomy for ampullary adenocarcinoma between January 2007 and December 2018 were retrospectively collected and analyzed.

**Results:**

The median number of lymph nodes per patient was 32 (20–46). Metastatic lymph nodes were found in 84 (76.4%) patients. In patients with positive nodules, the most commonly involved nodes were the #13 (80.1%) and #17 (78.6%) nodes, followed by #12 (69.0%) and #8 nodes (57.1%). Patients with 3–4 positive nodes among #13, #17, #12, and #8 had lower survival rates than those with 0 or 1–2 nodes.

**Conclusion:**

Ampullary adenocarcinoma commonly spreads to #13, #17, #12, and #8 lymph nodes. These nodes affected the patients' survival rates dramatically.

## Introduction

Ampullary adenocarcinoma is a rare type of cancer, accounting for 6% of periampullary neoplasms (1–4). Surgery gives rise to a better prognosis in ampullary adenocarcinoma patients than the one for pancreatic or biliary cancer (5–7). Variable factors such as resection margin, histologic type, invasion depth, perineural, and lymphatic invasion have been reported as predictors of a patient's prognosis. Because lymph node (LN) status is pivotal for ampullary adenocarcinoma (2, 8, 9), understanding the importance of lymphatic spread is critical for curative resection. Some studies reporting node metastasis have previously been published (9–11); however, due to the small study population in these studies (range 12–21), the importance of LN spread was not well known. Lymphadenectomy in patients with adenocarcinoma of the duodenal papilla increases surgical time, but not morbidity and mortality benefit, as well as hospital length of stay (12). However, the therapeutic benefit of the performance of a wider node dissection in patients with periampullary adenocarcinoma remains unclear (13). The purpose of this study was to identify the prognostic significance of positive nodes in ampullary adenocarcinoma.

## Methods

### Enrolled patients

The data of 121 ampullary adenocarcinoma patients who underwent operations from January 2007 to December 2018 in Shanghai Jiao Tong University Affiliated Sixth People's Hospital and Heilongjiang Provincial Hospital Affiliated to Harbin Institute of Technology were retrospectively collected based on a prospective database. Eleven patients were excluded due to R1 or R2 resection. The study population consisted of 70 patients who underwent the Whipple procedure and 40 patients who underwent the pylorus-preserving procedure (PPPD). All of the 110 patients underwent R0 resection. The demographics data, namely, age, gender, BMI, bilirubin, CA199, operative procedure, follow-up time, tumor size, invasion depth, differentiation, histological type, perineural invasion, lymphovascular invasion, resected lymph nodes, and positive lymph nodes, were collected and studied. The study was approved by the ethics committee of Shanghai Jiao Tong University. It was conducted following the principles of the Declaration of Helsinki. Each patient provided written informed consent.

### Surgical procedure

Curative resection was achieved when a negative resected margin was found, and all metastatic LNs were completely removed. All patients underwent surgery with standard regional lymphadenectomy including the peripancreatic LNs, the common and proper hepatic artery LNs, the LNs in hepatoduodenal ligament, and the LNs in the right lateral area of SMV (superior mesenteric vessel). However, the aortocaval and para-aortic LNs were only removed when they were found to be enlarged or positive in imaging before operation.

### Pathological examination

All adenocarcinomas originated from the ampulla of Vater. The histopathological examination of pancreaticoduodenectomy specimens was performed following the detailed standardized protocol (14). Tumor differentiation was ranked as well, moderate, and poor. The resected margin was evaluated. R0 resection was defined as the resected margins of the pancreas, common bile duct, duodenum, and retropancreatic tissues were free of cancer under the microscope. LNs were classified into groups and numbered following the General Rules for Surgical and Pathological Studies on Cancer of the Biliary Tract advised by the Japanese Society of Biliary Surgery (JSBS).

### Statistical analysis

Continuous variables were expressed as median and range or mean ± standard deviation, and categorical variables were expressed as number and percentage. The *χ*^2^ test was performed for nominal data. Univariate analysis was performed using the *χ*^2^ test or Fisher's exact test for categorical variables. When the data did not follow normal distributions, the non-parametric Mann–Whitney *U* test was applied. The Kaplan–Meier survival rate was compared using the log-rank test. Significant factors identified in univariate analysis were then subjected to multivariate analysis and were analyzed using Cox proportional hazard regression analysis. Significance was considered when *P*  < 0.05. SPSS 20 (SPSS, Chicago, IL, USA) was used for statistics.

## Results

### Patient demographics

The median follow-up time of the 110 patients was 40 (6–60) months (Table 1), and other clinical features are also shown in Table 1. There were 3,482 LNs resected from 110 patients were pathologically diagnosed. The median number of LNs per patient was 32 (20–46), 34 (20–42) for the Whipple procedure, and 27 (20–46) for the PPPD. Eighty-four (76.4%) patients had LN metastases. The median number of positive regional LNs was 4 (1–8).

**Table 1:** Characteristics of 110 patients of ampullary adenocarcinoma.

Patients (*n*)	110
Age (mean ± SD)	54.8 ± 11.4
Gender (F/M)	46/64
BMI (mean ± SD)	22.4 ± 2.2
Bilirubin (mg/dl) (median, range)	5.4 (0.4–23.3)
CA199 (U/ml) (median, range)	61.3 (2.2–1002.2)
Operative procedure, *n* (%)	
Whipple	70 (63.6%)
PPPD	40 (36.4%)
Follow up (months) (median, range)	40 (6–60)
Tumor size (cm) (mean ± SD)	3.2 ± 1.6
Invasion depth, *n* (%)	
T1	20 (18.2%)
T2	22 (20.0%)
T3	38 (34.5%)
T4	30 (27.3%)
Differentiation, *n* (%)	
Well	34 (30.9%)
Moderate	40 (36.4%)
Poor	36 (32.7%)
Histological type, *n* (%)	
Intestine	75 (68.0%)
Pancreatobiliary	35 (32.0%)
Perineural invasion, *n* (%)	50 (45.0%)
Lymphovascular invasion, *n* (%)	77 (70.0%)
Resected lymph nodes, *n* (median, range)	32 (20–46)
Positive lymph nodes, *n* (median, range)	4 (1–8)

### Distribution of LNs

The highest incidence of positive LNs was identified in posterior and anterior pancreaticoduodenum (#13, #17) (80.1%, 78.6%), followed by the LNs of the hepatoduodenal ligament (#12) (69.0%) and the common hepatic artery (#8) (57.1%) (Table 2). There were six patients with #8 or #12 LNs involved but without #13 or #17 LNs involved. The relationship between positive LNs and tumor invasion depth is shown in Table 3. The ratios of involved LNs at T1, T2, T3, and T4 were 20.0%, 63.6%, 78.9%, and 100.0%, respectively. The most common positive LNs were #13 and #17 LNs in all T stages, and the second most common positive LNs were #12 in the T2 stage, and #12 and #8 in both T3 and T4 stages.

**Table 2:** Distribution of positive lymph nodes in ampullary adenocarcinoma.

Group according to JSBS	Patient no. with positive nodes (%)
Perigastric (#7)	14 (16.7%)
Common hepatic artery (#8)	48 (57.1%)
Celiac trunk (#9)	11 (13.1%)
Hepatoduodenal ligament (#12)	58 (69.0%)
Posterior pancreaticoduodenal (#13)	68 (80.1%)
Anterior pancreaticoduodenal (#17)	66 (78.6%)
Superior mesenteric artery (#14)	7 (8.3%)
Para-aortic (#16)	2 (2.4%)

JSBS, the Japanese Society of Biliary Surgery.

**Table 3:** The correlation of positive LN with invasion depth.

Invasion depth (patient no.)	Patient no. with nodule involvement (%)	Involved LN (patient no.)	
		Most common	Second
T1 (20)	10 (20.0%)	#13 (8) and #17 (10)	
T2 (22)	14 (63.6%)	#13 (10) and #17 (8)	#12 (8)
T3 (38)	30 (78.9%)	#13 (30) and #17 (28)	#12 (30) and #8 (28)
T4 (30)	30 (100.0%)	#13 (30) and #17 (30)	#12 (30) and #8 (30)

LN, lymph node.

### Survival analysis

In univariate analysis, depth of invasion, LN status, tumor differentiation, perineural invasion, and lymphovascular invasion were strongly associated with the patients' 5-year overall survival rate after surgery (*P* < 0.01). A significant difference was found when the 5-year survival rate was compared between T1 + T2 and T3 + T4 tumors (75.3% vs. 36.4%, *P* < 0.01). Patients with negative LNs had a higher 5-year survival rate than those with positive LNs (71.3% vs. 42.3%, *P* < 0.01). There was a significant difference in the 5-year survival rates between well + moderate and poor differentiated tumors (64.3% vs. 44.5%, *P* < 0.01). Patients without perineural invasion or lymphovascular invasion had a higher 5-year survival rate than their counterparts (75.1% vs. 31.2%, *P* < 0.01 and 81.4% vs. 48.3%, *P* < 0.01) (Table 4). In the multivariate analysis, invasion depth (RR: 2.3, 95% CI: 1.6–3.4, *P* < 0.01), perineural invasion (RR: 1.9, 95% CI: 1.2–3.4, *P* < 0.01), lymphovascular invasion (RR: 2.6, 95% CI: 1.63–4.9, *P* < 0.01), LN status (RR: 14.5, 95% CI: 3.8–54.9, *P* < 0.01) and differentiation (RR: 2.5, 95% CI: 1.4–14.1, *P* < 0.01) were independently correlated with the patient's survival (Table 5).

**Table 4:** Univariate analysis of pathological variables on survival in patients with ampullary adenocarcinoma.

Parameters	Patient no.	5-year OS (%)	*P*-value
Invasion depth			
T1 + T2	42	75.3	
T3 + T4	68	36.4	<0.01
LN status			
Negative	26	71.3	
Positive	84	42.3	<0.01
Differentiation			
Well + moderate	74	64.3	
Poor	36	44.5	<0.01
Histological type			
Intestine	75	65.5	
Pancreatobiliary	35	62.3	NS
Perineural invasion			
Absent	55	75.1	
Present	50	31.2	<0.01
Lymphovascular invasion			
Absent	33	81.4	
Present	77	48.3	<0.01

NS, not significant, LN, lymph node, OS, overall survival.

**Table 5:** Multivariate analysis of variables on survival of ampullary adenocarcinoma patients.

Parameters	RR	95% CI	*P*-value
Invasion depth	2.3	1.6–3.4	<0.01
Perineural invasion	1.9	1.2–3.4	<0.01
Lymphovascular invasion	2.6	1.3–4.9	<0.01
Lymph node status	14.5	3.8–54.9	<0.01
Differentiation	2.5	1.4–14.1	<0.01

RR, relative risk, CI, confidence interval.

### Impact of the number of positive #13, #17, #12, and #8 LNs on patients' survival

**Figure 1:**
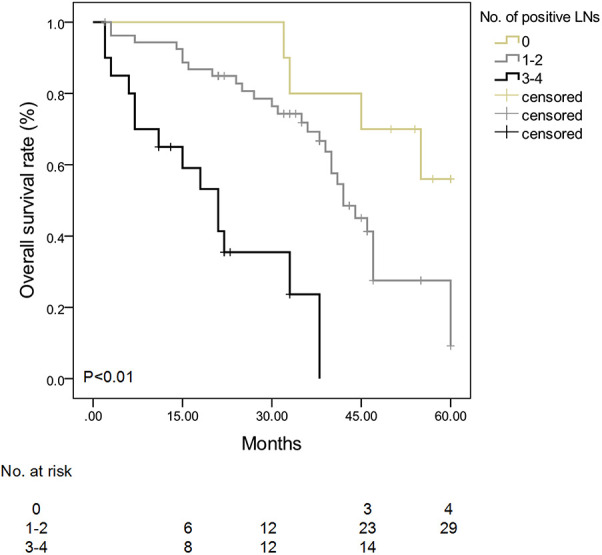
Survival curve stratified by the number of positive #13, #17, #12, and #8 lymph nodes in patients with ampullary adenocarcinoma. Patients with 3–4 positive nodes among #13, #17, #12, and #8 LNs had a lower survival rate than patients with 0 or 1–2 nodes (*P* < 0.01).

Patients with 3–4 positive nodes among the #13, #17, #12, and #8 LNs had a lower survival rate than patients with 0 or 1–2 nodes (*P* < 0.01) (Figure 1). In the multivariate analysis of LNs (#13, #17, #12, and #8) on patients' survival, patients with 1–2 positive nodes had a higher risk of death than those with negative nodes (RR: 3.5, 95% CI: 2.3–5.8, *P* < 0.01), and patients with 3–4 positive nodes had a higher risk of death as compared with patients with negative nodes (RR: 13.5, 95% CI: 3.1–44.5, *P* < 0.01) (Table 6).

**Table 6:** Multivariate analysis of lymph node (#13, #17, #12, and #8) status on survival in patients with ampullary adenocarcinoma.

Number of positive lymph nodes (among #13, #17, #12, and #8)	RR (95% CI)	*P*-value
0	1 (reference)	
1–2	3.5 (2.3–5.8)	<0.01
3–4	13.5 (3.1–44.5)	<0.01

RR, relative risk, CI, confidence interval.

## Discussion

The risk of LNs metastasis increases with tumor T stages as demonstrated by Lee et al. (6), Bronsert et al. (15, 16), and our study. Analysis based on the T stage seems to be more reasonable than other gross types. Delcore et al. found that tumor size was an independent factor for patient survival (17), but Berger et al. demonstrated that pancreatic invasion but not tumor size was the indicator for survival (18). Once the cancer cells penetrate the sphincter of Oddi, LNs are going to be involved. The invasion depth is a significant parameter for prognosis. Lee et al. found that the most commonly involved nodes were #13 LNs followed by #17 LNs. But no #12 and #8 LNs were involved. They suggested that the number of positive LNs, rather than their ratio and location, independently affected the patient's survival. They further found that the most common positive LNs were #13 and #17 LNs in the T1 stage. The #13 LNs were the most common positive LNs, and the #17 LNs were the second most common positive LNs in T2 to T4 stages. In this study, we found that the most common positive LNs were #13 and #17 LNs in all T stages, and the second most common positive LNs were #12 in the T2 stage and #12 and #8 in both T3 and T4 stages. The findings were quite different probably due to a relatively small patient number in Lee's study.

In the present study, the highest LN metastatic rates were seen in #13 and #17 LNs, followed by #12 and #8 LNs. The pattern of LN distribution found in our study is also quite different from other previous studies (10, 11). The #13 and #17 LNs were most frequently involved and had an increased incidence of involvement accompanied by T stages, suggesting that the #13 and #17 LNs were the initial and major metastatic locations. Kayahara et al. (10) suggested that the #13 LN was important for metastasis, and we also shared this opinion. Compared with the previous reports (9, 10, 17, 19), they showed that the occurrences of LN metastases to the perigastric region, common hepatic artery, and celiac trunk were rare. But we got the opposite result. We found that all T1 lesions had pancreaticoduodenal LNs (#13 and #17) metastasis while perigastric (#8 and #12) metastasis was exhibited only in T2, T3, and T4 tumors. This finding indicated that ampullary adenocarcinoma with pancreatic invasion seemed to be more like a pancreatic malignancy with a more extensive LN involvement (13).

Recently, the use of metastatic LN alone or the number of total LN in predicting prognosis may have a bias in the prognostic evaluation (18). The notion that the extensive regional LN dissection contributes to curative resection remains unproven. A randomized trial did not demonstrate the advantage of a more extensive regional node dissection (19). In this study, there is a significant difference in the survival between the numbers of positive #13, #17, #12, and #8 LNs, suggesting the advantage of regional LN dissection. Schwarz and Smith suggested that a pathological examination of a specimen should include at least 10 LNs (13). An inaccurate pathological process could miss positive LNs; therefore, the diagnosis from a skilled and experienced pathologist should be advocated. Hurtuk et al. reported a number of dissected LNs of 15 (2–38) (20). In our study, the median number of resected LN was 32 (20–46).

Due to the low prevalence of this disease, it is unlikely to conclude that the extended LN dissection will give rise to the patient's survival unless an adequate randomized trial with a sufficient sample size is introduced. However, we did find that the #13, #17, #12, and #8 LNs played a vital role in LN metastasis of ampullary adenocarcinoma which affected the patients' survival dramatically. It was necessary to perform a complete node resection in the areas of hepatoduodenal ligament and common hepatic artery in patients with ampullary adenocarcinoma, especially in T2, T3, and T4 lesions.

This study had some limitations concerning its retrospective nature. Although there were limitations, the new findings were remarkable and may contribute to this field in the following aspects. First, although the importance of LN metastasis on the prognosis of ampullary carcinoma was well established, this study demonstrated and confirmed the powerful importance of the analysis with a large number of LNs and more precisely described the pattern of LN distribution than previous reports. Second, according to the T stage, we described the spread pattern of LNs metastasis. This helps to decide the appropriate extension of LN dissection. Third, #13, #17, #12, and #8 LNs are so important that they need surgeon's attention during operation. The more LNs were dissected, the better prognosis would be achieved.

## Data Availability

The raw data supporting the conclusions of this article will be made available by the authors, without undue reservation.
